# Biosynthesis, characterisation and antimicrobial activity of zinc oxide and nickel doped zinc oxide nanoparticles using *Euphorbia abyssinica* bark extract

**DOI:** 10.1049/nbt2.12072

**Published:** 2021-11-26

**Authors:** Gezahegn Faye, Tola Jebessa, Tilahun Wubalem

**Affiliations:** ^1^ Department of Chemistry Jimma University Jimma Ethiopia; ^2^ Department of Chemistry Salale University Fiche Ethiopia; ^3^ Department of Chemistry Bule Hora University Bule Hora Ethiopia

**Keywords:** antimicrobial activity, biosynthesis, doping, *Euphorbia abyssinica*, XRD, ZnO NPs

## Abstract

Biosynthesis of metallic oxide nanoparticles is being used and preferred over physical and chemical methods of synthesis since it is simple, inexpensive, environmentally friendly, and green. The aim of this study was to synthesise ZnO and nickel doped ZnO nanoparticles using *Euphorbia abyssinica* bark extract for antimicrobial activity studies via agar disk diffusion method against some selected microbes. The synthesised nanoparticles were characterised using X‐ray diffraction (XRD), ultraviolet–visible spectroscopy, and Fourier transform infrared spectroscopy. The study results revealed that the biosynthesised nanoparticles had good crystalline nature, with crystal sizes in the range of nanoparticles and structures of hexagonal wurtzite. Both undoped ZnO and nickel doped ZnO nanoparticles demonstrated antibacterial and antifungal activity against four bacterial strains and two fungal genus. Generally, nickel doped ZnO NPs were found to possess more antimicrobial activities than undoped ZnO NPs. Specially, 4% and 5% nickel doped ZnO NPs showed significantly enhanced activity against *Enterococcus faecalis, Staphylococcus aureus, Aspergillus* and *Fusarium*.

## 1 INTRODUCTION

ZnO has high electron mobility, high thermal conductivity, good transparency and direct wide band gap of 3.37 eV. Moreover, ZnO is preferentially a stable hexagonal wurtzite, zinc blend and rock salt structure at room temperature and each oxygen ion surrounded by tetrahedral zinc ion [1]. ZnO has got remarkable research attention as a result of its innocent characteristics such as non‐toxicity or bio‐compatibility and stability as well as abundancy [2], and it also inhibits the action of pathogenic microbes when used in small concentration [3]. However, its wide band gap has limited the practical use of ZnO NPs as a photocatalyst and an antimicrobial agent. And doping of metals into ZnO NPs is receiving attention due to its improved properties and applications [4]. Fe [4], Mg [5], Ta [6] and Cu [7, 8] doped ZnO NPs with enhanced antimicrobial activities have been reported. The inhibitory mechanism of ZnO NPs is by the generation of Reactive Oxygen Species (ROS), metal ion release and membrane damage [9, 10, 11, 12, 13], and increase in the antimicrobial activities of metal doped ZnO have been mechanistically suggested due to the enhanced generation of ROS as a result of doping [4, 5, 6, 7, 8, 14]. However, a scholar suggests the requirement for further studies to exactly know the inhibitory mechanisms of the nanoparticles [9, 11].

Different classical methods of nanoparticle synthesis such as coprecipitation, thermal decomposition, hydrothermal, solgel, and electrochemical are known. However, they involve the use of hazardous solvents and apply expensive reagents [15]. Consequently, researchers have developed the green method for the synthesis of nanoparticles [16], which is an eco‐friendly method that uses minimum or no hazardous chemicals for the synthesis of nanomaterials thus receiving much attention [2, 15]. Many nanomaterials have been synthesised using biomaterials extracted from plants which are more efficient, inexpensive, eco‐friendly and of nontoxic approach [2]. Phytochemicals extracted from plants have been used for the synthesis of metal/metal oxide nanoparticles as reducing and stabilising agents [17], and *Aloe vera* [18], *Calotropis procera* [19], *Trifolium pratense* flower [3], and *Citrus aurantifolia* [20] extracts have been used for the synthesis of ZnO nanoparticles. *Amaranthus spinosus* leaf extract [4], *Prunus cerasifera* fruit extract [21] and *Cannabis sativa* leaf extract [14] have been used as reducing and stabilising agents for synthesis of Fe, Ag and Ag doped ZnO NPs respectively.

Traditionally, *Euphorbia abyssinica* plant parts have been used for curing fungal infection [22], cancer [23, 24], abdominal pain [25], hemorrhoid and skin wound [26], and malaria [27]. *Euphorbia abyssinica* bark has been also used by people traditionally for curing diseases like gonorrhea and syphilis [28]. It has been reported that the *Euphorbia abyssinica* bark plant extract contains metabolites like terpenoids, flavonoids, tri‐terpenoids and phenolic compounds [29]. However, to the best of our knowledge, there is no literature on the synthesis of ZnO and Ni doped ZnO nanoparticles using *Euphorbia abyssinica* plant extract. Thus, the main objective of this work was to synthesise ZnO and Ni doped ZnO nanoparticles by using *Euphorbia abyssinica* bark extract and to evaluate their antimicrobial activities.

## 2 MATERIALS AND METHODS

The chemicals and apparatus used during the process of synthesis include Nickel nitrate (Ni(NO_3_)_2_·6H_2_O), Zinc nitrate (Zn(NO_3_)_2_·6H_2_O), aluminium foil, DMSO, gentamicin, muffle furnace, 80 ml beaker, 250 ml beaker and ceramic crucible.

### 2.1 Plant sample collection and preparation

The bark of *Euphorbia abyssinica* (*Adaammi in Afaan Oromo)* was collected from Kiremu District, East Wollega, Oromia, Ethiopia. The collected *Euphorbia abyssinica* bark was washed with tap water thoroughly to remove debris and other contaminants, then dried in the sun shade and powdered using mortal and pistil. The maceration method of extraction was employed. 60 g of *Euphorbia abyssinica* bark was added to distilled water in a 300 ml beaker, and kept for 24 h for extraction. Then, the extract was filtered and stored for further experimental analysis.

### 2.2 Preparation of ZnO and Ni doped ZnO nanoparticles

The synthesis of ZnO nanoparticles was made by mixing bark extract (50 ml) and zinc nitrate hexahydrate (2 g) in a 75 ml beaker. The mixture was stirred well for 30 min using a magnetic stirrer. Then, the mixture was kept for 12 h for paste formation and this paste was collected in ceramic crucibles. The aforementioned ZnO NPs synthesis procedure was adopted for the synthesis of nickel doped ZnO NPs, and 1%–5% mole of nickel nitrate hexahydrate was added to the adjusted amount of zinc nitrate hexahydrate. The mass of zinc nitrate hexahydrate, the volume of extract and the calcination temperatures used were obtained by optimisation. All the products were calcinated for 2 h in a muffle furnace at 500°C and stored for characterisation. At the time of calcinations, the colour of the brown black samples changed to white in both ZnO and Ni doped ZnO samples.

### 2.3 Characterisation of ZnO and Ni doped ZnO nanoparticles

The synthesised ZnO and Ni doped ZnO nanoparticles were characterised by ultraviolet–visible spectroscopy (UV–Vis) absorption spectra in the wavelength range of 230–800 nm. The existence of functional groups left after calcination as capping and stabilisation agents were characterised using Fourier transform infrared spectroscopy (FTIR) spectra. The structural parameters of ZnO and nickel doped ZnO nanoparticles have been calculated from the X‐ray diffraction (XRD) pattern using Scherer equations [30].

### 2.4 Antimicrobial activity

Antimicrobial studies were conducted at the Jimma University in the Biology Department Laboratory and done using the agar disc diffusion method [30]. The biological screening effects of the synthesised nanoparticles were tested against the bacterial strains such as *Enterococcus faecalis, Staphylococcus aureus*, *Bacillus subtilis* and *Escherichia coli*. And fungal activities were also tested against *Aspergillus* and *Fusarium genuses*. Culture containing test tubes with approximately equal concentration or density of 0.5 McFarland standards [31] was used for inoculation of media. And freshly grown liquid culture of test bacteria and fungi solution having similar turbidity with 0.5 McFarland were seeded over Muller‐Hinton Agar (MHA) and Potato Dextrose agar respectively. DMSO was used as negative controls and ampicillin as positive. The sample solution of 20 mg/ml of each tested compound has been prepared by dissolving the compounds in DMSO and the solutions were loaded on the wells of the culture and in culture incubated at 37°C for 24 h. The inhibition zones were developed on the plate around the standard paper disc with adiameter of 6 mm, and the antimicrobial activity of nanoparticles was demonstrated by the diameter of the zone of inhibition developed around the sample.

## 3 RESULTS AND DISCUSSION

### 3.1 Phytochemical screening

The result of phytochemical screening showed that *Euphorbia abyssinica* plant bark extract possesses flavonoids, phenols, saponoids, steroids and tannin metabolites (Table 1). This finding agrees with the previous phytochemical screening results of *Euphorbia* species [32]. The existence of these secondary metabolites enables the *Euphorbia abyssinica* plant extract to be used as a reducing and stabilising agent during the synthesis of ZnO and Ni doped ZnO nanoparticles.

**TABLE 1:** Phytochemical screening of *Euphorbia abyssinica* bark extract

Number	Chemical constituent	Result
1	Alkaloids	‐
2	Flavonoids	+
3	Carbohydrates	+
4	Phenols	++
5	Saponoids	+
6	Steroids	+
7	Tannins	++

*Note*: ++, most presence; +, less presence; ‐, absent.

### 3.2 UV–visible spectral analysis

**FIGURE 1:**
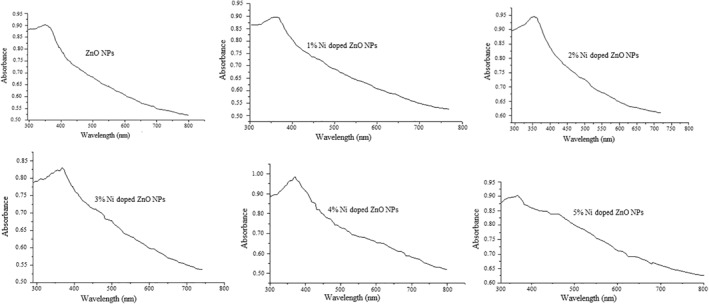
UV–visible spectra of ZnO and Ni doped ZnO nanocomposites

The amount of Zn(NO_3_)_2_·6H_2_O and the volume of the extract were optimised using the UV–visible spectra of ZnO nanoparticles synthesised from these two substances in different ratio. Based on the appearance of the peak and maximum wavelength, the optimum amount of Zn(NO_3_)_2_·6H_2_O and the volume of the extract used in the synthesis of ZnO were found to be 2 g and 50 ml respectively. For Ni doped ZnO nanoparticle synthesis, the amount of Ni(NO_3_)_2_·6H_2_O which was doped is indicated in Table 2. The UV–visible spectra of all the synthesised nanoparticles are indicated in Figure 1. The UV–visible spectra of the extract showed *λ*_max_ at 288 nm and that of zinc nitrate salt at 242 nm. The two peaks disappeared in the synthesised NPs and new peaks were observed that suggest the synthesis of ZnO and nickel doped ZnO NPs. The absorption peaks were observed in the wavelength range of 350–380 nm [33], and indicated the synthesis of ZnO and nickel doped ZnO nanoparticles. It is also strongly believed that the phytochemicals like phenols, phenolic, steroids and tannins are working as reducing agents and are responsible for conversion of metal salts into ZnO nanoparticles [17, 34].

**TABLE 2:** Maximum wavelength for ZnO and Ni doped ZnO nanoparticles

Amount of Ni doped (%)	Mass of Zn(NO_3_)_2_ (mg)	Mole of Zn(NO_3_)_2_ (mol)	Molarities of Zn(NO_3_)_2_ (M)	Mass of Ni(NO_3_)_2_ (mg)	Mole of Ni(NO_3_)_2_ (mol)	Molarities of Ni(NO_3_)_2_ (M)	Maximum wavelength (nm)	Volume of extract (ml)
0	2000	0.0067	0.134	0.00	0.00	0.00	351	50
1	1980	0.00666	0.133	19.5	0.00671	0.00134	352	50
2	1600	0.0054	0.017	38	0.00013	0.0026	355	50
3	1940	0.0065	0.13	58	0.00012	0.00392	366	50
4	1920	0.00645	0.129	78.5	0.00034	0.0054	368	50
5	1898	0.0064	0.127	99	0.00034	0.0068	369	50

### 3.3 Fourier transform infrared spectroscopy spectral analysis

**FIGURE 2:**
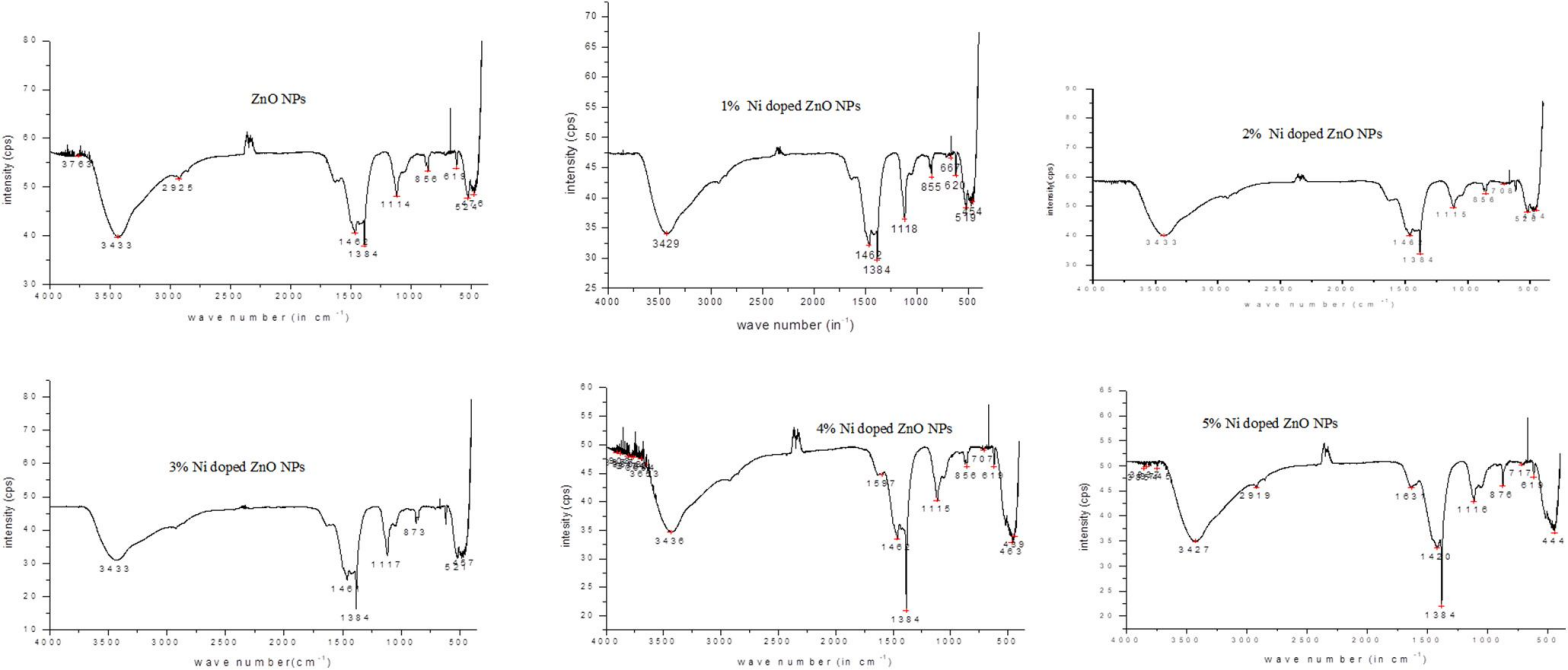
Fourier transform infrared spectroscopy spectra of ZnO and Ni doped ZnO NPs

Fourier transform infrared spectroscopy analysis confirms the presence of different organic and inorganic functional groups. The metal oxide absorption peaks range less than 1000 cm^−1^ [35]. FTIR result clearly indicated that the extract contains functional groups of alcohol, carboxylic acid, ether, phenols, tannins and ketone which may act as capping or stabilising agents for the formation nanoparticles [36]. The FTIR spectra (Figure 2) and the stretching of major functional groups presented in Table 3 are almost the same for all the synthesised NPs but there was some change in the wavelength noticed in Ni doped ZnO NPs. The stretching vibration observed at 524 cm^−1^ is due to Zn–O stretching, and this result is similar with the reported literature value of 530 cm^−1^ stretching vibration for Zn–O bond in Ni doped ZnO NPs [32].

**TABLE 3:** Fourier transform infrared spectroscopy spectra for ZnO and Ni doped ZnO NPs

Wave number in cm^−1^	Explanation for stretching/vibrations/bending
3427–3433	• O–H stretching vibration of hydroxyl groups in alcohol intermolecular bond and water
2850–2960	• C=C stretching of conjugated alkene
1597–1631	• C–H stretching vibration in alkane
1350–1450	• C–N, NO_3_^−^ stretching in amine and nitrate groups
1114–1117	• C–C stretching in alkane
817–873	• C=C bending in alkene
619–717	• Zn–O, Ni–O weak stretching in ZnO and NiO NPs
524	• Zn–O stretching vibration of ZnO NPs
444–457	• Ni–O stretching vibration of NiO NPs

### 3.4 X‐ray diffraction analysis

**FIGURE 3:**
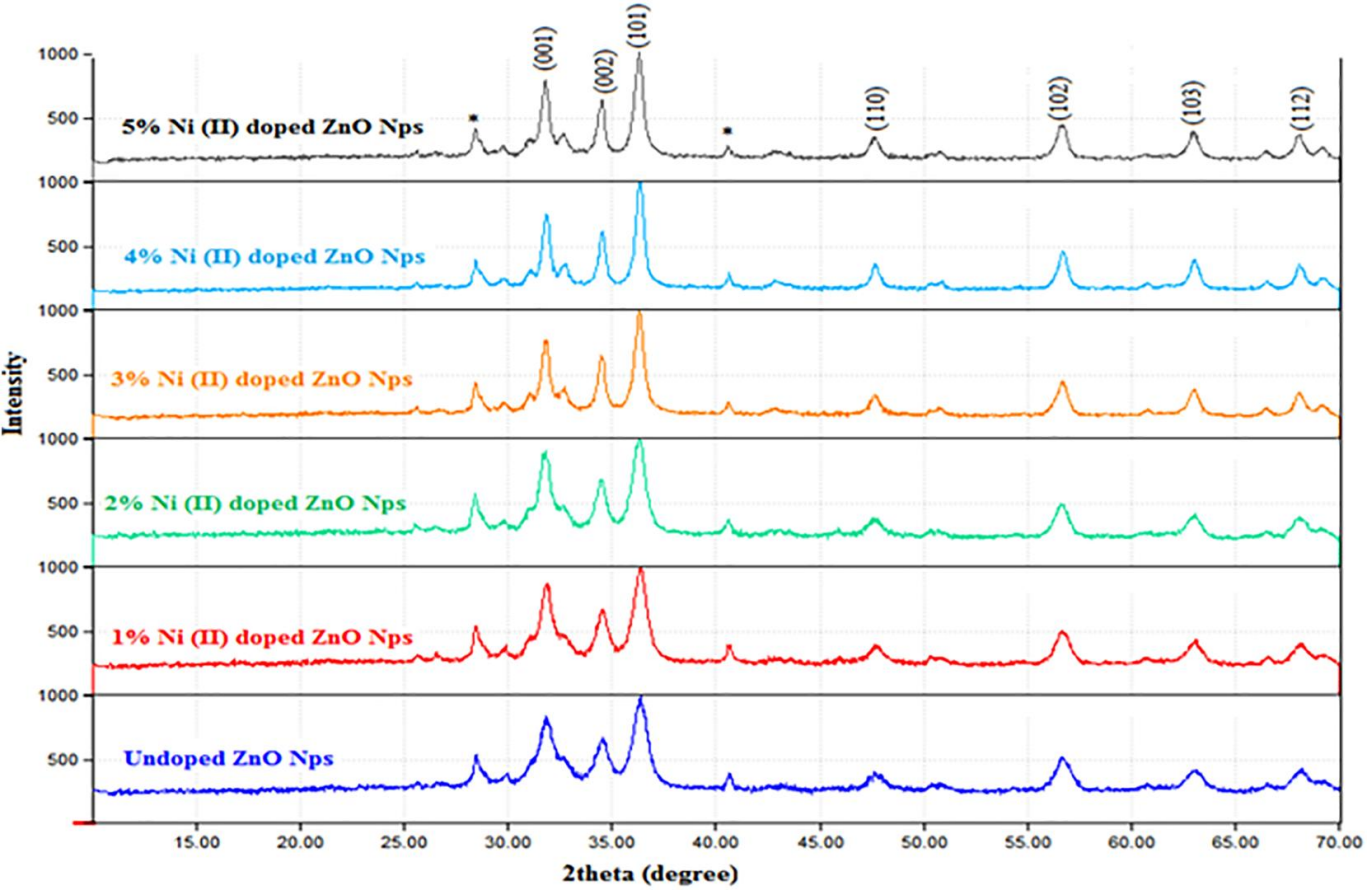
X‐ray diffraction spectra of ZnO and Ni doped ZnO NPs

X‐ray diffraction study result confirmed that the synthesised ZnO and Ni doped ZnO NPs were with hexagonal wurtzite phase (Figure 3 and Table 4) and the prominent peaks obtained correspond to the diffraction planes {001}, {100), {002},{101}, {102}, {110}, {103} and {112}, and are in good agreement with the study result of the ZnO nanoparticle synthesised using the *olea europaea* water extract [37]. The XRD data were recorded by using Cu Kα radiation (*λ* = 1.5406 Å). The average crystallite size (*D*) of ZnO and Ni doped ZnO NPs were calculated from the most intense peak (101) using the Debye Scherer formula (*D* = *kλ*/*β*_1/2_cos*θ*) [32, 38], where *k* denotes the Scherer constant *k* = 0.90 [39]*, λ* = 0*.1*5406 nm is the wavelength of the incident Cu *Kα* radiation; *β r*epresents the full‐width at half‐maximum (FWHM) of the respective peak. The diffraction peak of each sample was strong, sharp and narrow that indicates the good crystalline nature of biosynthesised ZnO and Ni doped ZnO NPs [40]. The size of the particles (Table 4) was found to be in the range of the nanoparticle material size of 1 to 100 nm [32, 41, 42].

**TABLE 4:** Crystal size calculated for ZnO and Ni doped ZnO NPs [intense peak (101) used]

Ni doped (%)	2*θ* (°)	Cos*θ*	*β*_1/2,_ FWHM (radian)	*D* = *kλ*/*β*_1/2_cos*θ* (nm)
0%	36.37	0.95	0.018	8.11 ± 1.32
1%	36.52	0.949	0.017	8.60 ± 1.29
2%	36.21	0.95	0.016	9.10 ± 1.33
3%	36.34	0.95	0.0105	13.89 ± 1.27
4%	36.36	0.95	0.0105	13.90 ± 1.34
5%	36.33	0.95	0.0098	14.89 ± 1.31

Abbreviation: FWHM, full‐width at half‐maximum.

Lattice constants *a*, *b*, and *c*, inter planar angle and unit cell volumes were calculated by using Bragg's equation, *λ* = 2*d*sin*θ* and Lattice geometry equation [36]. The lattice parameters were calculated by using sin*θ* = *λ*/2*a* (*h* ^2^ + *k* ^2^ + *l* ^2^)^1/2^, *a* = *λ*/(3)^1/2^sin*θhkl*, *c* = *λ*/sin*θ*, and *d*_*hkl*_ = 1/((4/3)^1/2^ (*h* ^2^ + *k* ^2^ + *hk*)/*a* ^2^ + *l* ^2^/*c* ^2^). Based on these formulas, the grain size or *d*‐spacing and *hkl* values were determined (Tables 5 and 6). When compared with the synthesised ZnO nanoparticles, there were no extra peak impurities observed in the synthesised Ni doped ZnO nanoparticles [43], and hence no significant change was observed in the hexagonal wurtzite structure of ZnO nanoparticles as a result of Ni doping. However, the diffraction angle at intensity (101) reflection of Ni doped ZnO NPs was slightly shifted relative to the diffraction angle 36.37° of ZnO nanoparticles which may indicate the doping of Ni into ZnO lattice and assures Ni is doped into ZnO nanoparticles [4, 41]. The bond length (*L*) was calculated by using *L* = ((*a* ^2^/3 + (1/2 − *u*)^2^_*C*_^2^)^1/2^, and the positional parameter (*u*) in the Wurtzite structure related to *c*/*a* ratio was determined by *u* = *a* ^2^/3*c* ^2^ + 0.25 equation. Positional parameter is the measure of the amount by which each atom is displaced with respect to ‘*c*’ axis. The increasing amount of dopant (Ni) from 1% to 5% resulted in the increase of particle size from 8.11 to 14.90 nm and decrease in the band gab from 3.53 to 3.46 eV, and the result was found to be similar with what was reported [4].

**TABLE 5:** Bandgap, grain size and unit cell parameters of ZnO and Ni doped ZnO NPs at miller index (101)

Synthesised nanoparticles	Grain size (nm)	Wavelength (nm)	Lattice parameters (Å)	*u* (KJ m^−3^)	*c*/*a*	Bandgap (eV)	*V* (Å^3^)
Undoped ZnO NPs	8.11 ± 1.32	351	*a* = 2.849	0.361	1.73	3.53	34.69
			*c* = 4.935				
1% Ni doped ZnO NPs	8.60 ± 1.29	352	*a* = 2.849	0.361	1.73	3.52	34.68
			*c* = 4.935				
2% Ni doped ZnO NPs	9.10 ± 1.33	355	*a* = 2.852	0.36	1.73	3.49	46.40
			*c* = 4.940				
3% Ni doped ZnO NPs	13.89 ± 1.27	356	*a* = 2.999	0.261	1.73	3.48	40.46
			*c* = 5.195				
4% Ni doped ZnO NPs	13.90 ± 1.34	357	*a* = 2.852	0.361	1.73	3.47	34.80
			*c* = 4.94				
5% Ni doped ZnO NPs	14.89 ± 1.31	358	*a* = 2.854	0.261	1.73	3.46	34.87
			*c* = 4.943				

**TABLE 6:** X‐ray diffraction spectrum of 5% Ni doped ZnO nanoparticles with their unit cell parameters

Peak number	2*θ* (°)	*d*	*a*	*c*	*h*	*k*	*l*
1	31.83	2.7927	3.245	5.62	0	0	1
2	34.50	2.5939	2.994	5.186	0	0	2
3	36.37	2.4698	2.849	4.935	1	0	1
4	47.96	1.9025	2.202	3.813	1	1	0
5	56.66	1.3383	1.874	3.246	1	0	2
6	62.96	1.4755	1.705	2.953	1	0	3
7	67.97	1.3383	1.598	2.751	1	1	2

### 3.5 Antimicrobial activity

**FIGURE 4:**
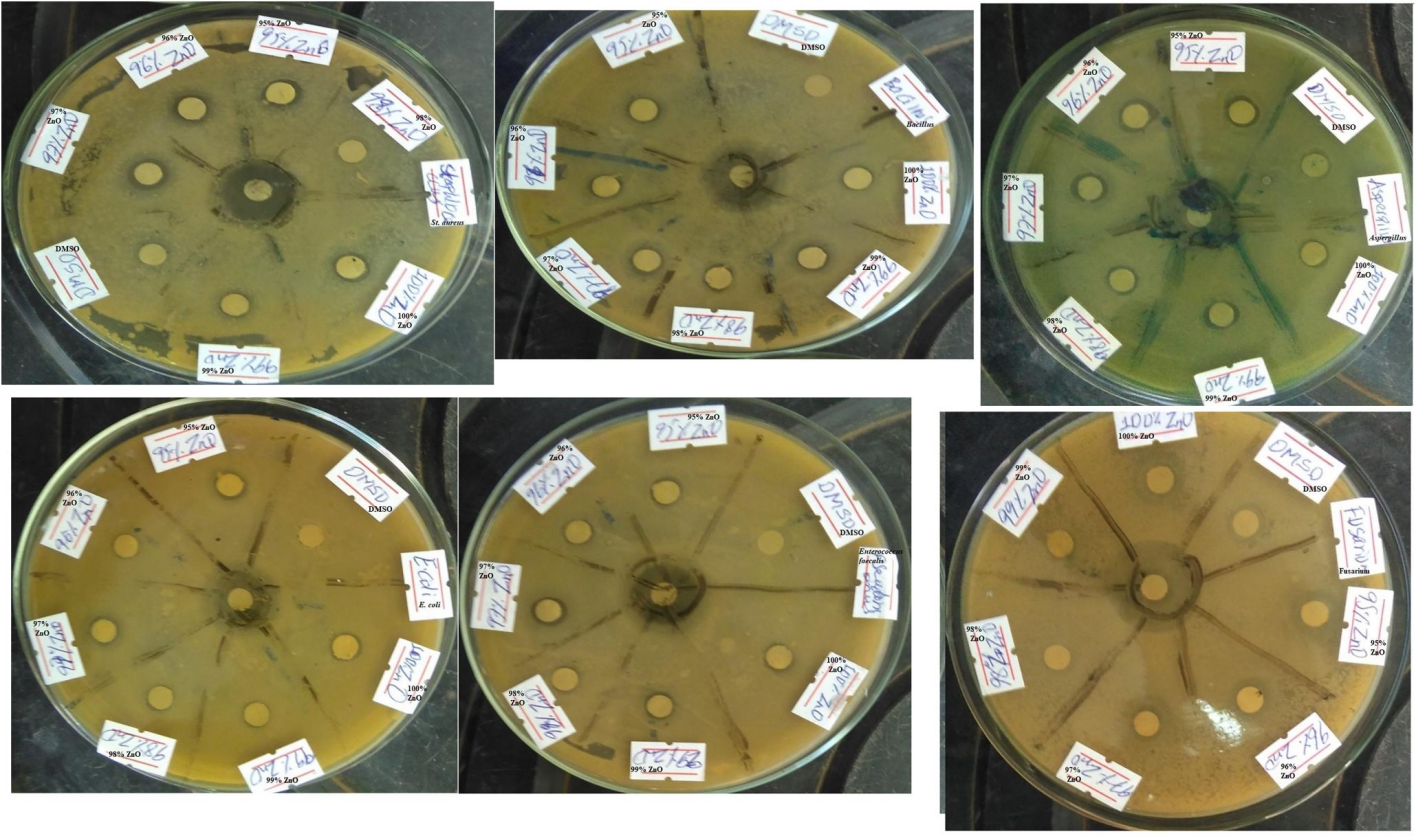
Diagram of zone of inhibition

The antimicrobial activities of ZnO and Ni doped ZnO NPs against *Enterococcus faecalis*, *Staphylococcus aureus, Bacillus subtitles, Escherichia coli, Aspergillus and Fusarium* were done using the disk diffusion method [42], and the diagram for the zone of inhibition is indicated in Figure 4. The antimicrobial activity study results of undoped ZnO, 1% Ni doped ZnO and 2% Ni doped NPs were found to be similar (Table 7) which is probably due to their close similarities in particle sizes (Tables 4 and 5). However, 4% and 5%, 4%, 5%, 4% and 5%, and 5% Ni doped ZnO nanoparticles showed significantly enhanced activity of 18%, 27%, 30%, 30% and 36% against *Enterococcus faecalis, Staphylococcus aureus, Bacillus subtitles, Aspergillus and Fusarium* respectively. Generally, Ni doped ZnO NPs showed improved antimicrobial activity when compared with undoped ZnO NPs, and this is supported in literature where doping of ZnO NPs with transition metals enhances its antimicrobial activity [44]. Enhanced antibacterial activity study results which are comparable with this study result was reported for Fe doped ZnO NPs synthesised by the green method using *Amaranthus spinosus* leaf extract [42] and for Ni and Co doped ZnO NPs synthesised by simple chemical co‐precipitation method [45].

**TABLE 7:** Antimicrobial activities of ZnO and Ni doped ZnO nanoparticles

Synthesised nanoparticles	*Staphylococcus aureus*	*Bacillus subtilis*	*Escherichia coli*	*Enterococcus faecalis*	*Aspergillus*	*Furasium*
Undoped ZnO	11 ± 0.11	10 ± 0.12	11 ± 0.11	11 ± 0.11	10 ± 0.14	11 ± 0.12
1% Ni and 99% ZnO	10 ± 0.12	9 ± 0.10	9 ± 0.13	10 ± 0.12	12 ± 0.10	11 ± 0.12
2% Ni and 98% ZnO	12 ± 0.13	12 ± 0.14	10 ± 0.13	12 ± 0.12	11 ± 0.13	10 ± 0.13
3% Ni and 97% ZnO	12 ± 0.21	12 ± 0.14	12 ± 0.10	12 ± 0.11	12 ± 0.11	12 ± 0.11
4% Ni and 96% ZnO	14 ± 0.13	12 ± 0.12	12 ± 0.14	13 ± 0.13	13 ± 0.12	13 ± 0.13
5% Ni and 95% ZnO	13 ± 0.14	13 ± 0.13	12 ± 0.12	13 ± 0.13	13 ± 0.12	15 ± 0.10
Gentamicin	19 ± 0.11	18 ± 0.12	16 ± 0.14	18 ± 0.11	18 ± 0.10	17 ± 0.12
DMSO	NI	NI	NI	NI	NI	NI

*Note*: Antimicrobial activity tests were done in triplicate (mean ± standard deviation); The measured zone of inhibition indicates significance differences (*p* < 0.05) according to ANOVA; 20 mg/ml each of ZnO and Ni doped ZnO NPs were used for antimicrobial activities.

## 4 CONCLUSION

ZnO and Ni doped ZnO NPs have been successfully synthesised by using *Euphorbia abyssinica* bark extract, and confirmed that this plant contains reducing and stabilisation agents, and could be used as potential agents for the biosynthesis of ZnO and Ni doped ZnO NPs. The characterisation results confirmed the synthesis of hexagonal wurtzite structure ZnO and Ni doped ZnO nanoparticles. As the amount of dopant (Ni) increases from 1% to 5%, the band gap was decreased and the particle size increased. Generally, 4% and 5% Ni doped ZnO NPs showed significantly enhanced antimicrobial activity when compared with the antimicrobial activity of the synthesised undoped ZnO NPs. Thus, the band gab and antimicrobial activity of ZnO NPs can be adjusted by using nickel as a dopant.

## CONFLICT OF INTEREST

The authors declare that they have no conflict of interest.

## Data Availability

All data generated or analysed during this study are included in this published article.

## References

[1] Ebrahimi, H.R. , Modrek, M. , Mozaffari, M. : Photo Degradation of Direct Red 81 (5‐Solamine) by using zinc oxide nanoparticles on glass granule substrate in acidic pH and various atmospheres. World Appl. Sci. J. 19(3), 352–354 (2012). 10.5829/idosi.wasj.2012.19.03.3007

[2] Rafaie, H.A. , Nor, R.M. , Amin, Y.M. : Magnesium doped ZnO nanostructures synthesis using *Citrus aurantifolia* extracts: structural and field electron emission properties. Mat. Express. 5(3), 226–232 (2015). 10.1166/mex.2015.1227

[3] Dobrucka, R. , Dugaszewska, J. : Biosynthesis and antibacterial activity of ZnO nanoparticles using *Trifolium pratense* flower extract. Saudi J. Biol. Sci. 23(4), 517–523 (2016). 10.1016/j.sjbs.2015.05.016 27298586 PMC4890195

[4] Aiswarya, S. , et al.: Strategy of metal iron doping and green‐mediated ZnO nanoparticles: dissolubility, antibacterial and cytotoxic traits. Toxicol. Res. 6(6), 854–865 (2017). 10.1039/c7tx00093f PMC6062353 30090548

[5] Pradeev raj, K. , et al.: Influence of Mg doping on ZnO nanoparticles for enhanced photocatalytic evaluation and antibacterial analysis. Nanoscale Res. Lett. 13, 229 (2018). 10.1186/s11671-018-2643-x 30076473 PMC6081874

[6] Guo, B.L. , et al.: The antibacterial activity of Ta‐doped ZnO nanoparticles. Nanoscale Res. Lett. 10, 336 (2015). 10.1186/s11671-015-1047-4 PMC4546064 26293495

[7] Carofiglio, M. , et al.: Doped zinc oxide nanoparticles: synthesis, characterization and potential use in nanomedicine. Appl. Sci. 10, 5194 (2020). 10.3390/app10155194 33850629 PMC7610589

[8] Khalid, A. , et al.: Synergistic effects of Cu‐doped ZnO nanoantibiotic against Gram‐positive bacterial strains. PLoS ONE. 16(5), e0251082 (2021). 10.1371/journal.pone.0251082 33989295 PMC8121369

[9] Maddahi, P. , et al.: Effect of doping on structural and optical properties of ZnO nanoparticles: study of antibacterial properties. Mater. Sci. Pol. 32(2), 130–135 (2014). 10.2478/s13536-013-0181-x

[10] Raufa, M.A. , et al.: Bougainvillea flower extract mediated zinc oxide’s nanomaterials for antimicrobial and anticancer activity. Biomed. Pharmacother. 116, 108983 (2019). 10.1016/j.biopha.2019.108983 31125822

[11] Al‐Shabib, N.A. , et al.: Biofabrication of zinc oxide nanoparticle from *Ochradenus baccatus* leaves: broad‐spectrum antibiofilm activity, protein binding studies, and in vivo toxicity and stress studies. Hindawi J. Nanomater. 2018, 1–14 (2018). 10.1155/2018/8612158

[12] Oves, M. , et al.: Anti‐microbial activity of cobalt doped zinc oxide nanoparticles: targeting water borne bacteria. J. Saudi Chem. Soc. 19, 581–588 (2015). 10.1016/j.jscs.2015.05.003

[13] Hussain, A. , et al.: Biogenesis of ZnO nanoparticles using *Pandanus odorifer* leaf extract: anticancer and antimicrobial activities. RSC Adv. 9, 15357–15369 (2019). 10.1039/c9ra01659g PMC9064228 35514831

[14] Chauhan, A. , et al.: Photocatalytic dye degradation and antimicrobial activities of Pure and Ag‐doped ZnO using *Cannabis sativa* leaf extract. Sci. Rep. 10, 7881 (2020). 10.1038/s41598-020-64419-0 32398650 PMC7217889

[15] Rajendran, S.P. , Sengodan, K. : Synthesis and characterization of zinc oxide and iron oxide nanoparticles using *Sesbania grandiflora* leaf extract as reducing agent. J. Nanosci. 2017, 1–7 (2017). 10.1155/2017/8348507

[16] Parthasarath, G. , et al.: Green synthesis of zinc oxide nanoparticles. World J. Pharm. Pharm. Sci. 5(4), 922–931 (2016). 10.20959/wjpps20164-6533

[17] Singh, J. , et al.: Green synthesis of metals and their oxide nanoparticles: applications for environmental remediation. J. Nanobiotechnol. 16, 84 (2018). 10.1186/s12951-018-0408-4 PMC6206834 30373622

[18] Sangeetha, G. , Rajeshwari, S. , Venckatesh, R. : Green synthesis of zinc oxide nanoparticles by *aloe* barbadensis miller leaf extract: structure and optical properties. Mater. Res. Bull. 46, 2560–2566 (2011). 10.1016/j.materresbull.2011.07.046

[19] Singh, R.P. , et al. : Biological approach of zinc oxide nanoparticles formation and its characterization. Adv. Mat. Lett. 2(4), 313‐317 (2011). 10.5185/amlett.indias.204

[20] Samat, N.A. , Nor, R.B.M. : Solgel synthesis of zinc oxide nanoparticles using *citrus aurantifolia* extracts. Ceram. Int. 39, 545–S548 (2013). 10.1016/j.ceramint.2012.10.132

[21] Ahmad, K.S. , Jaffri, S.B. : Phytosynthetic Ag doped ZnO nanoparticles: Semiconducting green remediators: photocatalytic and antimicrobial potential of green nanoparticles. Open Chem. 16(1), 556–570 (2018). 10.1515/chem-2018-0060

[22] Teklehaymanot, T. : Ethnobotanical study of knowledge and medicinal plants use by the people in Dek Island in Ethiopia. J. Ethnopharmacol. 124, 69–78 (2009). 10.1016/j.jep.2009.04.005 19477609

[23] Esubalew, S.T. , et al.: Review of ethnobotanical and ethnopharmacological evidences of some Ethiopian medicinal plants traditionally used for the treatment of cancer. Ethiop. J. Health Dev. 31(3) (2017)

[24] Abebe, W. : An overview of Ethiopian traditional medicinal plants used for cancer treatment. Eur. J. Med. Plants. 14(4), 1–16 (2016)

[25] Yemane, B. , Medhanie, G. , Surender Reddy, K. : Survey of some common medicinal plants used in Eritrean folk medicine. Am. J. Ethnomed. 4(2), 14 (2017). 10.21767/2348-9502.100014

[26] Mesfin, F. , Seta, T. , Assefa, A. : An ethnobotanical study of medicinal plants in Amaro Woreda, Ethiopia. Ethnobot. Res. Appl. 12, 341–354 (2014)

[27] Muluye, A.B. , et al.: Anti‐malarial activity of the root extract of *Euphorbia abyssinica* (*Euphorbiaceae*) against *Plasmodium berghei* infection in mice. Malar. J. 18, 261 (2019). 10.1186/s12936-019-2887-7 31362744 PMC6668069

[28] Geetha, M.S. , Nagabhushana, H. , Shivananjaiah, H.N. : Green mediated synthesis and characterization of ZnO Using *Euphorbia* Milli Latex as Fuel. Int. J. Sci. Res. 5(4), 158–163 (2016). 10.21275/v5i4.nov162504

[29] Prakash, M.J. , Kalyanasundharam, S. : Biosythesis, characterization of free radical scavenge ring activity and ant‐bacterial effects of plant‐mediated Zinc oxide nanoparticles using *Pithecellobium dulsce* and *Langeneria siceria* leave extract. World Sci. News 18, 100–117 (2015)

[30] Talam, S. , Karumuri, S.R. , Gunnam, N. : Synthesis, characterization, and spectroscopic properties of ZnO nanoparticles. ISRN Nanotechnol. 2012, 1–6 (2012). 10.5402/2012/372505

[31] National Committee For Clinical Laboratory Standards , Barry, A.L. : Methods for determining bactericidal activity of antimicrobial agents: approved guideline. vol. 19. National Committee for Clinical Laboratory Standards, Wayne (1999)

[32] Bhuiyana, M.R.A. , Rahman, M.K. , Rahman, M.K. : Synthesis and characterization of Ni doped ZnO nanoparticles. I.J. Eng. Manuf. 4(1), 10–17 (2014). 10.5815/ijem.2014.01.02

[33] Iqtedar, M. , et al.: Biosynthesis, optimization and characterization of ZnO nanoparticles using Bacillus cereus MN181367 and their antimicrobial activity against multidrug resistant bacteria. Rev. Mex. Ing. Quím. 19(Sup. 1), 253–266 (2020). 10.24275/rmiq/Bio1605

[34] Aluri, J. , Lakshmi, P.B., S. , Mandava, B.R. : Influence of Ni doping on structural and optical properties of ZnO nanopowders synthesised by sol–gel process. Sens. Transducers. 202(7), 66–71 (2016)

[35] Dubey, M. , Bhadauria, S. , Kushwah, B. : Green synthesis of nano silver particles from extracts *Eucalyptus hybrid* (safeda) leaf. Dig. J. Nanomater. Biostruct. 4, 537–543 (2009)

[36] Umaralikhan, L. , Jaffar, M.J.M. : Green synthesis of ZnO and Mg doped ZnO nanoparticles, and its optical properties. J. Mat. Sci. Mat. Electron. 28, 7677–7685 (2017). 10.1007/s10854-017-6461-1

[37] Hashemi, S. , et al.: Green synthesis of ZnO nanoparticles by Olive (*Olea europaea*). IET Nanobiotechnol. 10(6), 400–404 (2016). 10.1049/iet-nbt.2015.0117 27906141 PMC8676441

[38] Mariama, A.A. , et al.: Synthesis of NiO and Ni nanoparticles and their characterization. Digest J. Nanomater. Biostruct. 9(3), 1007–1019 (2014)

[39] Gonzalez, A.E.J. , Urueta, J.A.S. , Parra, R.S. : Optical and electrical characteristics of aluminum‐doped ZnO thin films prepared by sol–gel technique. J. Cryst. Growth 192(3), 430–438 (1998). 10.1016/S0022-0248(98)00422-9

[40] Mittal, A.K. , Chisti, Y. , Banerjee, U.C. : Synthesis of metallic nanoparticles using plant extracts. Biotechnol. Adv. 31(2), 346–356 (2013). 10.1016/j.biotechadv.2013.01.003 23318667

[41] Tharmar, T. , George, A.T. , Kumar, A.A. : Optical properties and FTIR studies of cobalt doped ZnO nanoparticles by simple solution method. Indian J. Sci. Technol. 9(1) (2016). 10.17485/ijst/2016/v9i1/85776

[42] Chauhan, J. , et al.: Synthesis and characterization of Ni and Cu doped ZnO. J. Nanomed. Nanotechnol. 8(2), 1–8 (2017). 10.4172/2157-7439.1000429

[43] Kermani, S. , Izadpanah, F. , Mirzaee, M. : The improvements in the size distribution of zinc oxide nanoparticles by the addition of a plant extract to the synthesis. Cogent Chem. 2(1), 1150389 (2016). 10.1080/23312009.2016.1150389

[44] Sharma, N. , et al.: Synthesis, characterisation and antimicrobial activity of manganese and iron doped zinc oxide nanoparticles. J. Exp. Nanosci. 11(1), 54–71 (2016). 10.1080/17458080.2015.1025302

[45] Ahmed, A.J. : Effect of transition metal doping on the structural, optical, thermal properties, and antimicrobial activity of zinc oxide nanoparticles. Int. Res. J. Pharm. 9(9), 16–24 (2018). 10.7897/2230-8407.099181

